# Real world data on the prognostic significance of monocytopenia in myelodysplastic syndrome

**DOI:** 10.1038/s41598-022-21933-7

**Published:** 2022-10-26

**Authors:** Panagiotis T. Diamantopoulos, Emmanouil Charakopoulos, Argiris Symeonidis, Ioannis Kotsianidis, Nora-Athina Viniou, Vassiliki Pappa, Charalampos Pontikoglou, Dimitrios Tsokanas, Georgios Drakos, Alexandra Kourakli, Elena Solomou, Eleftheria Hatzimichael, Anastasia Pouli, Maria Kotsopoulou, Evangelos Asmanis, Maria Dimou, Panayiotis Panayiotidis, Sotirios Papageorgiou, Georgios Vassilopoulos, Achilles Anagnostopoulos, Theodoros Vassilakopoulos, Helen Papadaki, Athanasios Galanopoulos

**Affiliations:** 1 Hematology Unit, First Department of Internal Medicine, Laikon General Hospital, National and Kapodistrian University of Athens, “Laikon” General Hospital, 11527 Athens, Greece; 2 Department of Internal Medicine, University Hospital of Patras, Rio, Greece; 3 Department of Hematology, University Hospital of Alexandroupolis, Alexandroupoli, Greece; 4 Haematology Division, Second Department of Internal Medicine, Attikon General Hospital, National and Kapodistrian University of Athens, Athens, Greece; 5 Haematology Laboratory, School of Medicine, University of Crete, Crete, Greece; 6 Department of Clinical Hematology, “G. Gennimatas” District General Hospital, Athens, Greece; 7 Department of Hematology, University of Ioannina, Ioannina, Greece; 8 Department of Hematology, St. Savvas Oncology Hospital, Athens, Greece; 9 Department of Hematology, Metaxa Anticancer Hospital, Piraeus, Greece; 10 Hematology Unit, First Department of Propedeutic Medicine, Laikon General Hospital, National and Kapodistrian University of Athens, Athens, Greece; 11 Department of Hematology, Larissa University Hospital, University of Thessalia, Larissa, Greece; 12 Hematology Department, General Hospital of Thessaloniki “George Papanikolaou”, Thessaloniki, Greece; 13 Department of Hematology, Laikon General Hospital, National and Kapodistrian University of Athens, Athens, Greece

**Keywords:** Leukaemia, Myelodysplastic syndrome

## Abstract

Monocytopenia is a common finding in patients with myelodysplastic syndrome (MDS), but although monocytes may exhibit prognostic significance in MDS due to their role in innate immunity, they have not been incorporated in any prognostic scoring system for MDS. In this study, we analyzed national registry data from 1719 adults with MDS. Monocytopenia was present in 29.5% of the patients and was correlated with the presence of excess blasts and higher revised international prognostic scoring system categories. Univariate analysis showed that monocytopenia was prognostic of a lower overall survival [(OS), 32.0 versus 65.0 months, p < 0.001], while it retained its prognostic significance in a multivariate model comprising anemia, neutropenia and thrombocytopenia [hazard ratio (HR) for OS, 1.320, p < 0.001]. Moreover, it was prognostic of a lower leukemia free survival (LFS) both in univariate analysis and in a multivariate model comprising cytopenias, bone marrow blasts, and cytogenetic risk (HR for LFS 1.27, p = 0.031). The findings regarding OS and LFR were exclusive or more pronounced in lower risk patients, respectively. Moreover, monocytopenia could divide the low and intermediate risk groups of IPSS-R in prognostically distinct subgroups. Our results redefine the prognostic role of monocytes in MDS and set the basis for further studies to validate our results and expand our knowledge on the prognostic significance of monocytopenia in MDS.

## Introduction

The prognosis of patients with myelodysplastic syndrome (MDS) is currently based on the International Prognostic Scoring System (IPSS)^1^ introduced in 1997, the revised IPSS (IPSS-R)^2^, and the World Health Organization (WHO) Classification-Based PSS (WPSS)^3^ that followed a few years later. Little is known for the prognostic significance of monocytopenia and none of the above prognostic systems considers its potential prognostic role, although monocytopenia is a common finding in MDS and the monocytes may participate in the prognosis of MDS as part of the innate immune response. They function to regulate cellular homeostasis, especially in the setting of infection and inflammation^4^ and account for approximately 5% of circulating nucleated cells in normal adult blood with a half-life of approximately 1–3 days.

In the present study, we evaluated the prognostic significance of monocytopenia in patients with MDS registered in a retrospective registry for MDS (Hellenic National MDS registry).

## Methods

We analyzed clinical and laboratory data from patients with MDS diagnosed per the 2008 WHO classification and recorded in a large retrospective national registry. Data were gathered during a 6-month period. The study comprised adult patients diagnosed with MDS per the 2008^5^ or the 2016 WHO classification^6^ who had survival data available for analysis. Patients with MDS/myeloproliferative neoplasms (MPN) and/or acute myeloid leukemia (AML) were excluded from the analysis. Patients with monocytosis were also excluded from the analysis, in order to avoid including patients with chronic myelomonocytic leukemia in the cohort. Moreover, patients eventually treated with allogeneic hematopoietic cell transplantation were censored for overall survival (OS) and leukemia-free survival (LFS). Baseline data included epidemiologic characteristics (gender, age) and hematologic parameters at diagnosis (hemoglobin, absolute neutrophil count, absolute monocyte count, platelet count, bone marrow and peripheral blood blast percentage, number of cytopenias). Blood counts were derived from automated complete blood counts. The baseline cytogenetic results were interpreted per the International System for Human Cytogenetic Nomenclature (ISCN 2005)^7^, while the cytogenetic risk was calculated for both the IPSS and IPSS-R. The patients were categorized per the IPSS, IPSS-R, and WPSS for MDS. Treatment data were also available. The OS rate was defined as the time interval from diagnosis to death from any cause. AML transformation was also recorded and analyzed. The study was designed and carried out by the Hellenic MDS Study Group, which is a Scientific Division of the Hellenic Society of Hematology.

Statistical analysis was performed using the IBM Statistical Package for Social Sciences (SPSS) statistics, version 23.0 (IBM Corporation, North Castle, NY, USA). The Pearson Chi-Square test was run to determine relationships between categorical variables and the Independent-Samples Mann–Whitney U test to check relationships between a categorical variable with two levels and not normally distributed continuous variables. Kaplan–Meier analysis were performed to estimate LFS and OS. Multivariate cox regression models were used, including variables that proved to be statistically significant in the univariate analysis. Median values and a 95% confidence interval were used in the analysis. The level of significance for all statistical tests was set at a probability value lower than 5% (2-sided p < 0.05). All methods were carried out in accordance with relevant guidelines/regulations.

### Ethics approval and consent to participate

This study was approved by the ethics committee of the Hellenic Society of Hematology and the ethics committee of the University Hospital of Patra, Rio, Greece. Informed consent was waived by the ethics committee of the University Hospital of Patra, Rio, Greece.

## Results

### General patient characteristics and definition of monocytopenia

The study comprised 1719 patients with MDS the main characteristics of whom are shown in Table 1. At the time of data cut-off, 818 patients were deceased and the median follow-up for the remaining 901 patients was 23.0 months.

**Table 1:** Patient characteristics.

Characteristic	Result
Number of patients at diagnosis, N (%)	1719 (100)
Male:female	1.89
Age at diagnosis (years), median (range)	74.0 (18.0–97.0)
**IPSS group at diagnosis, N (%)**	
Low	756 (44.0)
Intermediate 1	614 (35.7)
Intermediate 2	255 (14.8)
High	94 (5.5)
**IPSS-R group at treatment initiation, N (%)**	
Very low	401 (23.3)
Low	667 (38.8)
Intermediate	273 (15.9)
High	229 (13.3)
Very high	149 (8.7)
Hemoglobin (g/dL), median (range)	9.7 (3.9–15.8)
Absolute neutrophil count (× 10^9^/L), median (range)	2.15 (0.0–26.5)
Platelet count (× 10^9^/L), median (range)	158 (0–846)
Absolute monocyte count (× 10^9^/L), median (range)	0.3 (0.00–0.99)
Absolute monocyte count < 0.2 × 10^9^/L, N (%)	507 (29.5)
**BM blast percentage, N (%)**	
< 5	1201 (69.9)
5–10	291 (16.9)
11–20	227 (13.2)
**Cytogenetic risk score (IPSS), N (%)**	
Low	1283 (74.6)
Intermediate	271 (15.8)
High	165 (9.6)

### Correlation of monocytopenia with baseline characteristics of the cohort

The median absolute monocyte count (AMC) was 0.30 × 10^9^/L (0.00–0.99 × 10^9^/L), and monocytopenia, defined as an AMC below 0.2 × 10^9^/L in the peripheral blood, was present in 507 (29.5%) of the patients. Patients with excess blasts (RAEB1/2) tended to have lower AMC (median 0.19 versus 0.32 for patients without excess blasts, p < 0.0001) and lower AMC were found in higher IPSS-R categories (very low, 0.37 × 109/L; low, 0.30 × 109/L; intermediate, 0.25 × 109/L; high, 0.16 × 109/L; very high, 0.20 × 109/L), while there was a highly significant difference in the AMC between higher risk (intermediate, high, very high) and lower risk (very low and low) MDS according to the IPSS-R (0.21 × 10^9^/L vs 0.33 × 10^9^/L, p < 0.0001).

### Prognostic significance of monocytopenia (whole cohort)

**Figure 1:**
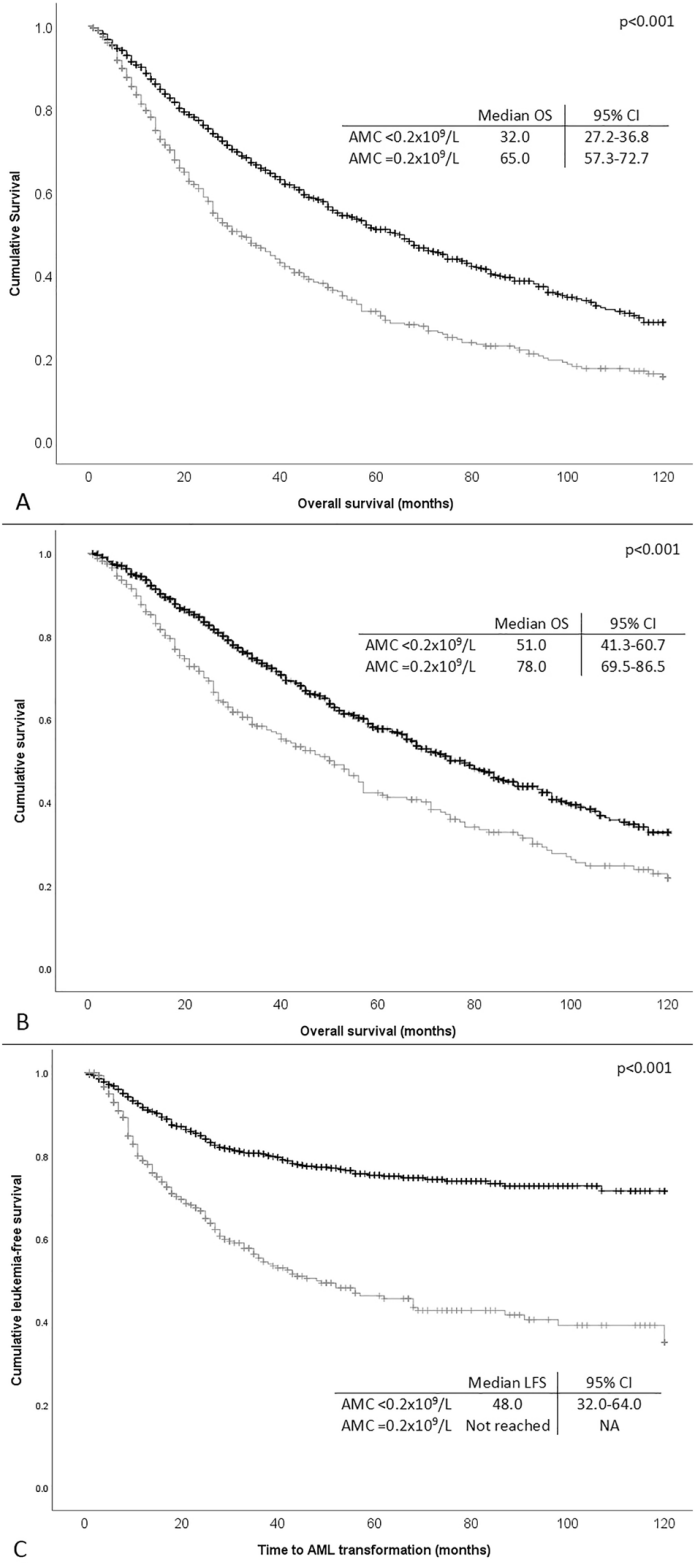
Kaplan–Meier curves estimating (**A**) overall survival (OS) of the whole cohort of 1719 patients with and without monocytopenia (absolute monocyte count < 0.2 × 10^9^/L), (**B**) OS of patients with lower (low and intermediate 1) risk score per the IPSS with and without monocytopenia, and (**C**) leukemia free survival of the whole cohort of patients with and without monocytopenia.

In univariate analysis, patients with an AMC below 0.2 × 10^9^/L were characterized by a median OS of 32.0 (95% CI 27.2–36.8) months as opposed to 65.0 (95% CI 57.3–72.7) months for patients with AMC over 0.2 × 10^9^/L (p < 0.001, Fig. 1A). Monocytopenia maintained its prognostic significance in a multivariate regression analysis which included a hemoglobin level below 10 g/dL, an absolute neutrophil count (ANC) below 0.8 × 10^9^/L, and a platelet number below 100 × 10^9^/L (all of them being predictive for OS in univariate analysis) [hazard ratio (HR), 1.320; 95% CI 1.134–1.537, p < 0.001]. Detailed data are presented in Table 2A. A positive correlation between the AMC and the ANC (Pearson Correlation 0.393, p < 0.0001) could be identified. Nonetheless, in a model comprising of neutropenia and monocytopenia, both parameters were independently correlated to OS. In addition, in a Cox regression analysis including AMC below 0.2 × 10^9^/L, the cytogenetic risk score per the IPSS-R, the number of cytopenias, and bone marrow blasts (classified per the IPSS-R), no further prognostic value was observed for AMC (HR 1.04; 95% CI 0.89–1.21; p = 0.643).

**Table 2:** Cox regression analysis model comprising monocytopenia (AMC < 0.2 × 10^9^/L), anemia (hemoglobin < 10 g/dL), neutropenia (ANC < 0.8 × 10^9^/L) and thrombocytopenia (PLT < 100 × 10^9^/L).

Covariate	Hazard ratio	95% CI	*p* value
**(A) Overall survival—whole cohort**			
Anemia	2.044	1.760–2.373	< 0.001
Neutropenia	1.266	1.045–1.535	0.016
Thrombocytopenia	1.782	1.532–2.072	< 0.001
Monocytopenia	1.320	1.134–1.537	< 0.001
**(B) Overall survival—lower risk (IPSS) patients**			
Anemia	2.020	1.695–2.409	< 0.001
Neutropenia	1.135	0.866–1.488	0.358
Thrombocytopenia	1.560	1.285–1.894	< 0.001
Monocytopenia	1.310	1.311–1.580	0.005
**(C) LFS—whole cohort**			
Anemia	1.641	1.318–2.044	< 0.001
Neutropenia	1.585	1.218–2.062	0.001
Thrombocytopenia	2.123	1.703–2.647	< 0.001
Monocytopenia	1.912	1.528–2.394	< 0.001
**(D) LFS—whole cohort**			
Bone marrow blast score (IPSS)	3.857	3.225–4.614	< 0.001
Cytogenetic risk score (IPSS)	3.432	2.633–4.474	< 0.001
Cytopenia score (IPSS)	2.556	1.606–4.069	< 0.001
Monocytopenia	1.420	1.135–1.776	0.002

A. The model was applied for OS in the whole cohort. B. The model was applied for OS only in lower risk patients per the IPSS. C, D. The models were applied for LFS in the whole cohort. All variables were found to be prognostic of a lower OS/LFS in univariate analysis. *OS* overall survival, *LFS* leukemia free survival, *CI* confidence interval, *IPSS* international prognostic scoring system.

### Prognostic significance of monocytopenia in different IPSS risk categories

After stratification per the IPSS categories, low AMC was prognostic for low OS only in patients with lower (low and intermediate 1) IPSS score [median OS, 51.0 (95% CI 41.3–60-7) months for patients with low AMC vs 78.0 (95% CI 69.5–86.5) months for those with high AMC, p < 0.001, Fig. 1B). Monocytopenia retained its prognostic significance in a Cox regression analysis model also comprising anemia, neutropenia and thrombocytopenia in this group of patients (Table 2B). Nevertheless, there was no additional prognostic impact in a model comprising cytopenias, cytogenetic risk group, and bone marrow blast count.

Moreover, monocytopenia was prognostic for LFS, since patients with low AMC (< 0.2 × 10^9^/L) had a median LFS of 48.0 months, while the median LFS for patients with higher AMC was not reached (p < 0.001, Fig. 1C). The prognostic significance of monocytopenia for LFS was maintained in a multivariate Cox regression analysis comprising hemoglobin below 10 g/dL, ANC below 0.8 × 10^9^/L, and platelet counts below 100 × 10^9^/L (HR 1.912; 95% CI 1.528–2.394, p < 0.001, details in Table 2C), all of which were prognostic for OS in univariate models. In a Cox regression model including the above stated factors (cytopenias, bone marrow blasts, cytogenetic risk, and monocytopenia), monocytopenia retained its prognostic significance for LFS (HR 1.27; 95% CI 1.02–1.58; p = 0.031, Table 2D). Again, in the subgroup of patients with lower IPSS score, low AMC was correlated with lower LFS (120 months versus “not reached” for patients with high AMC, p < 0.001).

### Incorporation of monocytopenia in the IPSS-R

We moreover tried to incorporate monocytopenia as a variable in IPSS-R. For that purpose, we assigned 0 points to patients without monocytopenia and 0.5 points to patients with monocytopenia (arbitrarily, following the grading of neutropenia used in IPSS-R). Thus, summing up the points of all variables, the minimum sum would be 0 and the maximum 10.5 (instead of 10 in the original IPSS-R). Then we updated the risk groups of IPSS-R counting monocytopenia along with the remaining cytopenias, keeping the original categorization of IPSS-R (i.e. very low, ≤ 1.5; low, 2.0–3.0; intermediate, 3.5–4.5; high, 5.0–6.0; very high, > 6.0). Restratification resulted in updated distinct “IPSS-R-monocytopenia” categories, and analysis with Kaplan–Meier curves revealed that the updated IPSS-R could stratify the cohort as shown in sFigure 1B.

**Figure 2:**
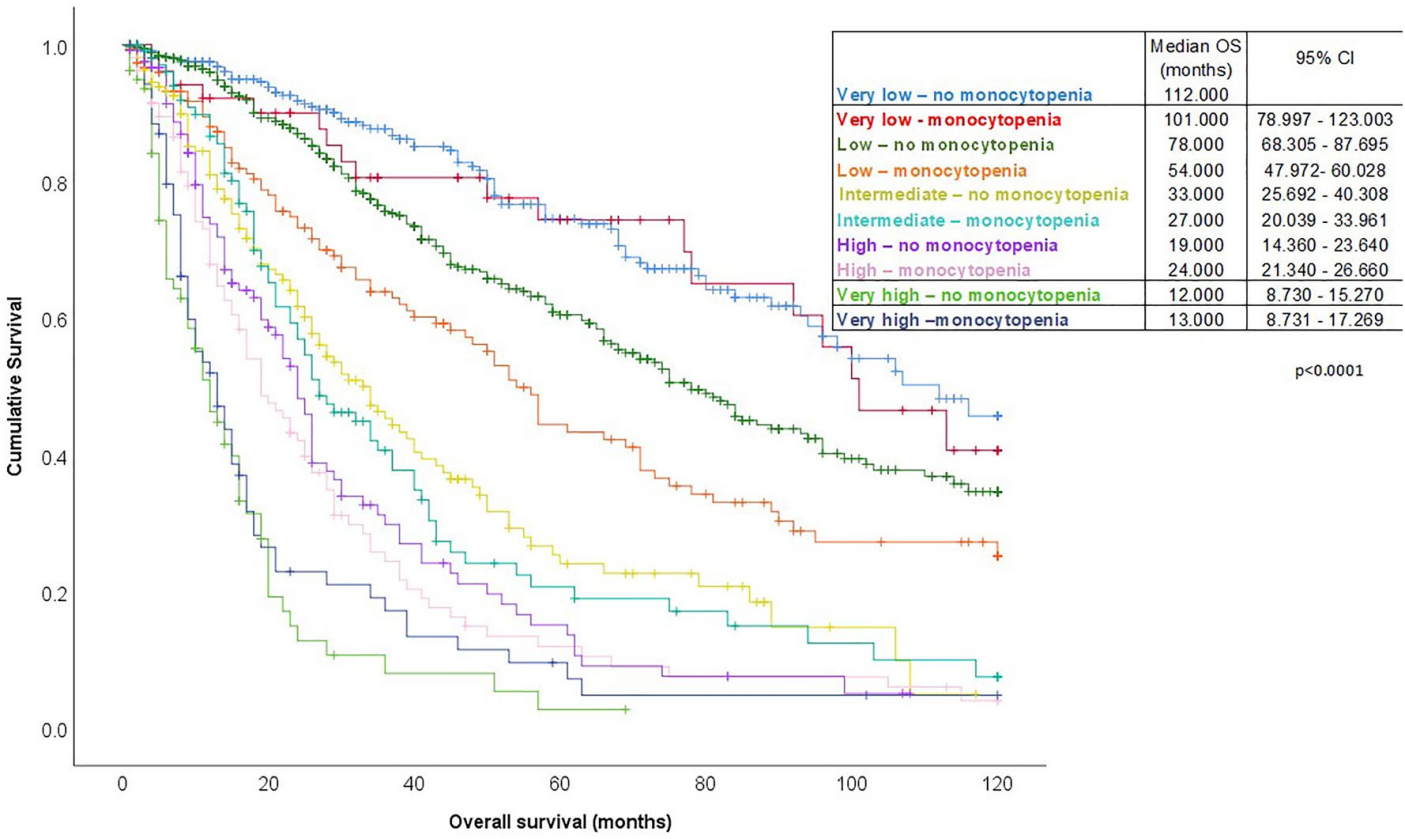
Kaplan–Meier curve estimating overall survival (OS) of the whole cohort of patients according to IPSS-R, but restratified using monocytopenia as an adjunct factor in each of the five risk groups of IPSS-R. As shown in the graph, ten new risk groups have emerged (each of the IPSS-R risk groups with and without monocytopenia). It is evident that especially in low and intermediate risk patients, monocytopenia can divide these risk groups in prognostically distinct subgroups.

Finally, using the original IPSS-R, we divided each risk group in two subgroups based on the monocyte count (one with and one without monocytopenia). This way, ten instead of five risk groups emerged (i.e. very low with and very low without monocytopenia and so on). Kaplan–Meier analysis revealed that patients with low, and intermediate risk per the IPSS-R were successfully restratified in subgroups of different median OS as shown in Fig. 2.

### Monocytopenia in patients treated with HMAs

In a subgroup of 162 patients treated with hypomethylating agents (HMAs), monocytopenia was not predictive of response to treatment, but low AMC was correlated to a shorter median progression free survival (27.0 months vs not reached for patients with higher AMC, p = 0.001). This correlation was not translated into a survival benefit (survival after HMA initiation, 27.0 vs 28.0 months respectively, log rank p = 0.213).

## Discussion

Monocytopenia can result from several conditions such as chemotherapy induced myelosuppression, hairy cell leukemia, aplastic anemia, hemodialysis, HIV infection, corticosteroid administration^8^ and in the MonoMAC syndrome due to haploinsufficiency of GATA2 (located at 3q21.3) leading to GATA2 deficiency that is characterized by disseminated nontuberculous mycobacterial infections or disseminated fungal disease^9^. Although monocytopenia has been associated with impaired granuloma formation it does not necessarily reflects low tissue macrophages, but irrespective of that, it has been correlated with increased susceptibility to several infections. Moreover, it can indicate poor prognosis in patients with acetaminophen-induced hepatitis^10^ and thermal injuries^11^. Nevertheless, the prognostic significance of monocytopenia is usually disregarded since in most cases it occurs along with neutropenia.

In MDS, the prognostic role of monocytopenia has been scarcely discussed in a few studies, with no definitive results. There are only two published studies on the prognostic significance of monocytopenia in MDS. The first one studied the prognostic significance of lymphocytopenia and monocytopenia in 889 patients with MDS, as well as the prognostic significance of lymphocyte-to-monocyte ratio (LMR). The investigators found that among the above mentioned variables, a high LMR (≥ 5) was the only parameter retaining its statistical significance as a prognostic factor for lower OS in multivariate analysis including other risk factors, while monocytopenia, although prognostic of a lower OS in univariate analysis, did not retain its significance in multivariate analysis. No correlations were found for LFS^12^. Applying the concept of LMR to our cohort, we found that an LMR ≥ 5 was associated with a lower OS [median OS, 43.0 months (95% CI 37.8–48.2) versus 67.0 months (95% CI 57.6–76.5) for patients with an LMR < 5]. Nevertheless, LMR lost its statistical significance in a multivariate model comprising anemia, thrombocytopenia, and neutropenia, while in the same population low AMC retained its prognostic significance. Thus, our study does not support the prognostic significance of LMR.

The second study was based on the Düsseldorf MDS-registry and comprised 976 patients. Once again monocytopenia was correlated with lower OS but only in the univariate analysis in the whole cohort. Nevertheless, after stratification per the IPSS-R, an independent prognostic value of monocytopenia was documented only in intermediate risk patients^13^. In our study no such correlation was evident in the intermediate risk group (median OS 48.8 months for low AMC versus 50.6 months for high AMC, p = 0.541).

Based on a large patient cohort, we found that patients with MDS with excess blasts as well as higher risk patients per the IPSS-R have low AMC. Moreover, we showed that low AMC is prognostic of low OS in univariate analysis and of low LFS in both univariate and multivariate analysis, highlighting a possible pathogenetic role for monocytopenia in MDS. The prognostic significance of low AMC seems to be stronger in lower risk patients, while in higher risk patients their prognostic role may be lost due to the effects of antineoplastic treatments such as the use of HMAs. Nevertheless, in patients treated with 5-azacytidine, monocytopenia, although not correlated with treatment response, it was correlated with a shorter progression free survival.

Incorporating monocytopenia as a variable in IPSS-R showed that the updated prognostic model still stratifies our cohort. Validation in other cohorts is still needed. Finally, and most importantly, patients with low, and intermediate risk per the IPSS-R can be restratified in subcategories with distinct prognosis when using monocytopenia as an adjunct factor. This finding is extremely important since patients within an IPSS-R risk group can be subdivided in distinct prognostic groups, with potential treatment implications. For example, low risk patients with monocytopenia have a median OS of 54 months versus 78 months for low risk patients without monocytopenia. A similar dichotomization of the intermediate risk group can be achieved using monocytopenia as an adjunct risk factor. Thus, patients with monocytopenia in these groups may need to be treated more aggressively (probably with HMAs) than patients without monocytopenia. It should be noted though, that monocytopenia loses its prognostic impact in very low, high, and very high risk patients. Validation of our results in other cohorts will help further define the prognostic role of monocytopenia in patients with MDS and decide whether monocytopenia should be incorporated as a variable in IPSS-R.

Moreover, it is worth mentioning that the correlation of monocytopenia with leukemic transformation provides new information on another aspect of the role of monocytes in the pathogenesis of MDS. In melanoma and ovarian cancer animal models, monocytes have been shown to participate in cancer immune surveillance by clearing cancer cells, consequently inhibiting metastatic expansion^14–16^. Thus, opposite to what previously was speculated, i.e. that monocytopenia conferred to the shortening of the survival of patients with MDS because of its implication in infectious processes, it may be speculated that monocytopenia may have a pathogenetic role per se in the progression of the disease.

The strengths of the present study are the large group of well characterized patients with MDS with no admixture of MDS/MPN or AML in the study group, as well as the fact that it deals with a poorly studied parameter in MDS. Thus, it provides novel, useful information on the prognostic significance of monocytopenia in MDS that could be used to formulate new, more accurate prognostic scores. The limitations of the study are its retrospective nature and its inability to prove a definitive correlation with survival in multivariate analysis. Nevertheless, it is well-known that single hematological parameters are not strong independent prognostic factors for OS in MDS. Further analysis is needed to define the exact prognostic role of AMC in MDS and its potential for incorporation to the current prognostic scoring systems for MDS.

## Conclusions

In summary, we showed that monocytopenia, as defined by an AMC < 0.2 × 10^9^/L, represents a dismal prognostic factor in MDS in the low and intermediate 1 IPSS risk groups independently of the prognostic impact of anemia, thrombocytopenia, and neutropenia. Moreover, our results suggest that monocytopenia is predictive of a lower LFS in MDS, even when taking into consideration the effect of cytopenias, the number of bone marrow blasts, and the cytogenetic risk group. Monocytopenia was also shown to divide the low and intermediate IPSS-R risk groups in two prognostically distinct subgroups (those with and without monocytopenia). Finally, we demonstrated that monocytopenia is correlated to a shorter median progression free survival in patients treated with HMAs. Overall, this study offers valuable information regarding the role of monocytes in the prognosis of MDS and paves the way for future research towards incorporation of monocytopenia into established prognostic scores in MDS.

## Supplementary Information

Supplementary Information 1.

## Data Availability

The data that support the findings of this study are available upon reasonable request from the corresponding author, PD. The data are not publicly available due to their containing information that could compromise confidentiality of patient health records.

## Abbreviations

AMC: Absolute monocyte count
AML: Acute myeloid leukemia
ANC: Absolute neutrophil count
HMA: Hypomethylating agent
HR: Hazard ratio
IPSS: International prognostic scoring system
IPSS-R: Revised international prognostic scoring system
LFS: Leukemia free survival
LMR: Lymphocyte to monocyte ratio
MDS: Myelodysplastic syndrome
MPN: Myeloproliferative neoplasm
OS: Overall survival
SPSS: Statistical package for the social sciences
WHO: World Health Organization
WPSS: World Health Organization Classification-based Prognostic Scoring System
