# Supplementary figures and images for: Real world data on the prognostic significance of monocytopenia in myelodysplastic syndrome
Source: Sci Rep. 2022 Oct 26;12:17914. doi: 10.1038/s41598-022-21933-7 (PMC9606364; doi:10.1038/s41598-022-21933-7)

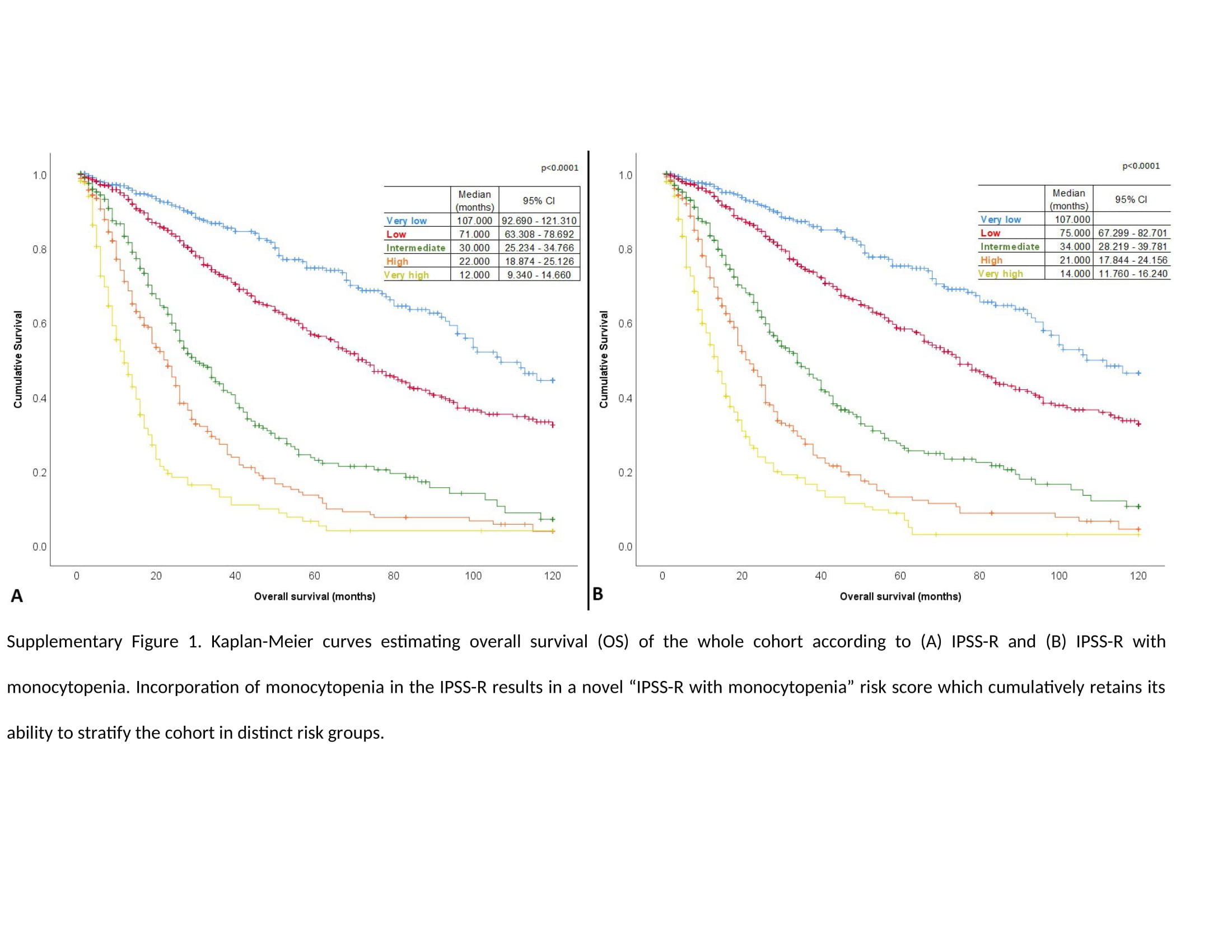

Supplement: Supplementary file 1 — Supplementary Information 1. [file 41598_2022_21933_MOESM1_ESM.jpg]
